# What about Dinner? Chemical and Microresidue Analysis Reveals the Function of Late Neolithic Ceramic Pans

**DOI:** 10.3390/molecules26113391

**Published:** 2021-06-03

**Authors:** Jaromír Beneš, Valentina Todoroska, Kristýna Budilová, Jaromír Kovárník, Jaroslav Pavelka, Nevenka Atanasoska, Jiří Bumerl, Assunta Florenzano, Tereza Majerovičová, Václav Vondrovský, Michaela Ptáková, Petr Bednář, Lukáš Richtera, Lukáš Kučera

**Affiliations:** 1 Laboratory of Archaeobotany and Palaeoecology, Faculty of Science, University of South Bohemia, Na Zlaté stoce 3, 370 05 České Budějovice, Czech Republic; benes.jaromir@gmail.com (J.B.); krr.budilova@gmail.com (K.B.); jkovarnik@prf.jcu.cz (J.K.); nevenka_atanasoska@yahoo.com (N.A.); jbumca@gmail.com (J.B.); tmajerovicova@gmail.com (T.M.); mdivisova@seznam.cz (M.P.); 2 Institute of Archaeology, Faculty of Philosophy, University of South Bohemia, Branišovská 31, 370 05 České Budějovice, Czech Republic; 3 Kej 8 Noemvri br.24/6, 6330 Struga, North Macedonia; t.valentina23@gmail.com; 4 Centre of Biology, Geosciences and Environmental Education, University of West Bohemia, Univerzitni 8, 30614 Plzen, Czech Republic; japetos@cbg.zcu.cz; 5 Laboratory of Palynology and Paleobotany, Department of Life Sciences, University of Modena and Reggio Emilia, via G. Campi 287, 41125 Modena, Italy; assunta.florenzano@unimore.it; 6 Institute of Archaeology of the Czech Academy of Sciences, 118 01 Prague, Czech Republic; vaclav.vondrovsky@gmail.com; 7 Department of Analytical Chemistry, Faculty of Science, Palacký University, 17. Listopadu 12, 779 00 Olomouc, Czech Republic; petr.bednar@upol.cz; 8 Department of Chemistry and Biochemistry, Mendel University in Brno, Zemědělská 1, 613 00 Brno, Czech Republic; richtera@mendelu.cz; 9 Central European Institute of Technology, Brno University of Technology, Technická 123, 612 00 Brno, Czech Republic

**Keywords:** archaeobotany, ceramic vessel, cholesterol, pests, phytoliths, starch, proteins, gas chromatography

## Abstract

The Late Neolithic palafitte site, Ustie na Drim, in the northern part of Lake Ohrid (North Macedonia), excavated in 1962, offered ceramic fragments of large, flat, elongated pans. These artifacts could be dated by relative chronology to roughly around 5200–5000 BC. According to their shape and technological traits, the ceramic pans were probably used for baking. The attached materials on the surface of studied pan fragments were sampled for consequent chemical and microscopical analyses (i.e., analyses of starch, phytoliths, and microscopic animal remains). An immunological method revealed the presence of pork proteins in samples. The presence of organic residues of animal origin was, moreover, confirmed by the detection of cholesterol using gas chromatography coupled to mass spectrometry. Analysis of detected microscopic botanical objects revealed starch grains of several plants (i.e., oak, cattail, and grasses). An interesting find was the hair of a beetle larva, which could be interpreted contextually as the khapra beetle, a pest of grain and flour. Based on our data, we suppose that the ceramic pans from Ustie na Drim were used for the preparation of meals containing meat from common livestock in combination with cereals and wild plants.

## 1. Introduction

One of the major topics in the contemporary bioarchaeology of artifacts is the investigation of archaeological vessels using the latest instrumental methods of chemical research and the recent tools of archaeobotanical, genetic and microbiological investigation. An analysis of the shape, quality of material, and volume of a found vessel enables an estimation of its use in the initial living context before being discarded and its existence during cultural deposition [1]. It is also possible to record the ‘life of artifacts’ from the time of their production and the beginning of their use to the moment of their exclusion. Today great attention is given to the residual content of vessels in Mediterranean regions due to chemical signals in residue content on the inner surface of vessels [2,3,4,5,6]. Analogically, ceramic vessels from the Iron Age in Central Europe have been subjected to chemical research that has revealed the presence of several compounds indicating food remains [7]. Besides ceramic vessels, special interest has been focused on analyzing the content of bronze vessels where organic residues could be ‘trapped’ in corrosion products [8,9]. The aim of the research on archaeological vessels is to identify their original function in society and contribute to an understanding of the subsistence principles as well as ritual customs of past human populations [2,10].

The Neolithic period in the Near East, Anatolia [11,12], and Europe [13] could be characterized by the movement of people and the diffusion of new modes of life. However, it should be noted that the development of populations in the Neolithic period was long and diverse [14]. Variability in their forms of settlement was also reflected in the development of ceramics: from a relatively simple ceramic type in the Early Neolithic to more complex variability in the Late Neolithic/Eneolithic. Current research on the functional traits of Neolithic pottery is targeted towards organic bulk remains on the inner surface of ceramics as well as chemical signals of organic penetration in the microporous matrix of ceramic vessels [15,16]. 

Detailed chemical analysis of soil content and/or organic residues in ceramic vessels and pans can provide information about their usage and former content. The analysis of lipid residues present therein is an important part of this research due to the chemical stability of nonpolar compounds in archaeological contexts (i.e., lipids, steroids, and terpenes) [17,18]. A frequently used technique for the characterization and identification of fat origins is the analysis of isotope ratios of individual fatty acids adsorbed in prehistoric, antiquity, and medieval ceramics using gas chromatography-combustion-isotope ratio mass spectrometry (GC-C-IRMS) [19,20]. However, ‘conventional’ gas chromatography combined with mass spectrometry (GC/MS) can provide similar information, and sometimes even more significant, when the use of the multiple-reaction monitoring method (MRM transition) is compared to GC-C-IRMS. The use of GC/MS together with MRM transition has been applied in the analysis of soil extracts from Neolithic ceramic vessels where a broomcorn millet marker miliacin has been found. The obtained results have improved our knowledge concerning the use of millet in the past and can be highlighted as the first direct evidence of usage of broomcorn millet in Central Europe [21]. Another technique for the analysis of original and intact lipid molecules, i.e., di- and tri-acylglycerols, is matrix-assisted laser desorption/ionization mass spectrometry [22,23]. As mentioned above, the stability of nonpolar compounds is much higher than the stability of polar compounds in an archaeological context due to the higher level of hydrolysis and water leaching of polar compounds [17,18]. For example, sterols, such as cholesterol in animals and ß-sitosterol in plants, are reasonably resistant to post-burial degradation and can therefore be a marker of fat origin [9,24].

Until recently, only a few research teams have worked with the chemical signals and archaeobotanical micro-objects from the residual material of the inner walls of archaeological vessels [16,25,26,27]. The aim of this article was to combine advanced chemical analysis (gas chromatography/mass spectrometry and immunological analysis) with microscopic evidence of the microremains (starch grains, phytoliths, molds, yeast cells, and other microremains) to determine the function of ceramic pans from the Late Neolithic palafitte site of Ustie na Drim in the Lake Ohrid shore area in the town of Struga, North Macedonia. 

## 2. Results and Discussion

**Figure 1:**
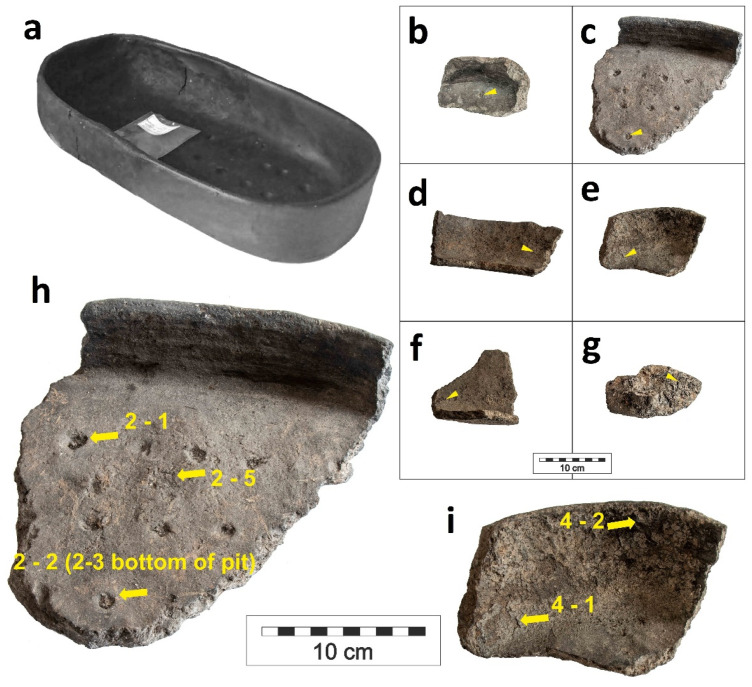
Reconstructed ceramic pan KE7 (**a**), sampled fragments KE1 (**b**), KE2 (**c**), KE3 (**d**), KE4 (**e**), KE5 (**f**), KE6 (**g**), and significant samples from KE2 and K4 ((**h**,**i**), respectively).

**Figure 2:**
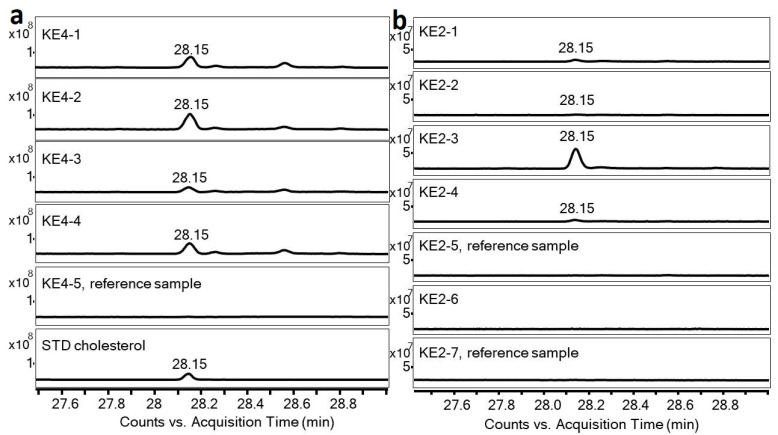
GC chromatogram of cholesterol in samples taken from ceramic pans KE4 (**a**) and KE2 (**b**).

The gas chromatography/mass spectrometry of organic residues attached on the surface of ceramic pans KE1–KE7 revealed a cholesterol signal (Table 1, Figure 1). The presence of cholesterol in all samples was confirmed by the authentic cholesterol standard, retention time, and fragmentation spectrum (Figure 2). The most significant sample was from ceramic pan KE4, where a high amount of a thin, baked mass was found. The concentrations of cholesterol in the sample taken from this thin, baked layer on the bottom of ceramic pan KE4-1 was 0.44 mg·g^−1^, while in the sample under the inner edge of vessel KE4-2, it was 0.66 mg·g^−1^; for the sample under the residue of sample KE4-1 (i.e., KE4-3) it was 0.19 mg·g^−1^, and in the sample of the mass under sample KE4-2 (i.e., KE4-4) it was 0.46 mg·g^−1^ (Figure 2a). The reference sample from the edge of vessel KE4 contained only traces of cholesterol (below 0.01 mg·g^−1^). This significant difference in the concentration of cholesterol in the KE4 samples excludes the cross-contamination of pan pits by surrounding material at the storage location. The samples from KE4 were also analyzed using immunological tests for the detection of denatured proteins. This methodology has been successfully tested many times [28,29] and was also used as a control for mass spectrometric data [23]. The sample KE4-2, with a high concentration of cholesterol, provided a positive reaction for porcine proteins. However, a positive reaction was also found in the reference sample KE4-5. Based on these results, contamination tests were performed for porcine proteins [29]. The contamination tests pointed to different origins for the porcine proteins in samples KE4-2 and KE4-5. The proteins in sample KE4-2 had been denatured by high temperatures compared with the ‘unchanged’ (undenatured) proteins in KE4-5. The results obtained by immunological analysis confirmed different chemical compositions of both examined layers. Moreover, a signal of 18-norabietane (RT 16.65 min.) and retene (18.64 min.) was found in samples KE4-2 and KE4-4, pointing to the presence of resin/decayed wood [30]. Note that these compounds were also found in other samples, and in the reference samples, the content of 18-norabietane and retene was 32 times and 8 times lower, respectively, than that of sample KE4-2. The total ion current (TIC) chromatogram of other compounds identified in sample KE2-4 is shown in Supplemental Figure S1 and Table S1.

**Figure 3:**
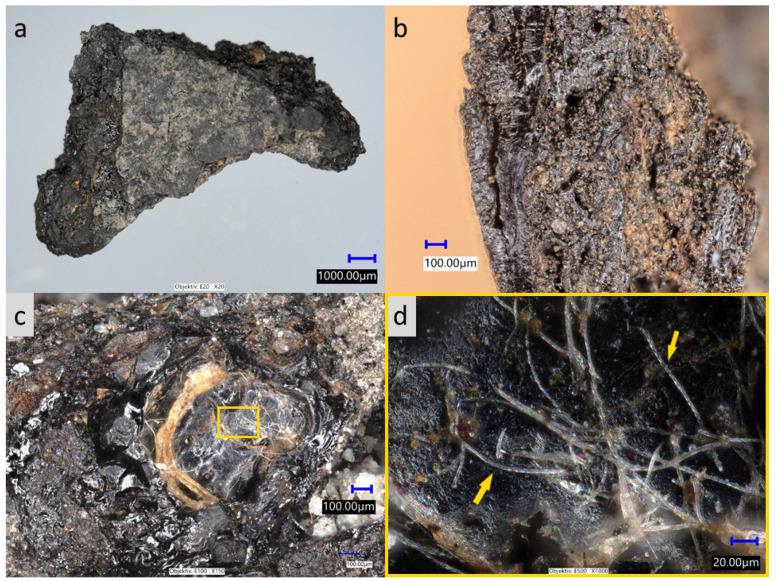
Ustie na Drim. Organic mass taken from ceramic pan KE7 before reconstruction. (**a**) profile of organic mass, (**b**) profile detail—the glassy edge burn-on, and (**c**,**d**) glassy mass with fungi hyphae baked inside.

The highest concentration of cholesterol was found in the pit of pan KE2 (Figure 1h, Figure 2b), i.e., 0.92 mg·g^−1^. Other samples taken from KE2 contained only a trace amount of cholesterol (i.e., the reference sample from the surface, KE2-7, 0.01 mg·g^−1^; sample of ceramic from the pit, KE2-2, 0.01 mg·g^−1^; sample of ceramic from the pit, KE2-4, 0.04 mg·g^−1^). Note that in the second reference sample (KE2-5, sample from the bottom of the pan) and in sample KE2-6, cholesterol was not detected. This significant difference in concentration of cholesterol in KE2 samples excludes the possibility of cross-contamination of ceramic pans’ pits by the surrounding material at the storage location. In ceramic pan KE1, the highest concentration of cholesterol was found in a sample taken from a pit similar to ceramic pan KE2; however, in the reference sample KE1-3, cholesterol was not detected. Besides the samples from pits, the organic residues attached to the bottoms and edges of ceramic pans KE3-KE6 were also analyzed. The concentration of cholesterol in these samples was 3–66 times higher compared to the appropriate reference sample (Table 1). Finally, baked organic mass (Figure 3) associated with the reconstructed ceramic pan (KE7) was analyzed by GC/MS. Cholesterol was found at a concentration of 0.17 mg·g^−1^. Abietic acid was also detected, but due to the fact that a suitable reference sample was unavailable, we did not try to interpret this. In this organic mass, baked remains of fungi hyphae have been observed through the glassy mass (Figure 3c,d). 

The samples from KE4-2 and KE7 were dated using the AMS radiocarbon method. Comparing both resulting dates, we can see that they are statistically inconsistent at the 5% significance level (T = 7.9, T(5%) = 3.8, df = 1). Though the calibrated probability distributions partially intersect, the KE 7 sample was earlier than sample KE 4-2 (Table 2). Both dates significantly contradict the archaeological chronology of the site (ca. 5200–5000 BC), and we, therefore, considered them unreliable, particularly because the carbonized food residues adhering to pottery have been proved to be generally problematic material for radiocarbon dating. Due to their heterogeneous composition, it is difficult to remove all sources of exogenous carbon [31]. Analyses of replicate measurements have evinced that dating food residues can produce inaccuracies of between 15% and 30% in the results, and offsets between replicate measurements can reach more than 1000 radiocarbon years [32,33]. As both radiocarbon dates from the Ustie na Drim site had approximately the same deflection from the expected chronology, we should also consider that they may have been influenced by a reservoir/hard water effect from Lake Ohrid [34], for which the modern rate has been estimated at ca. 1500 radiocarbon years [35]. Despite the sampled food residues being of terrestrial origin (see above), it should be noted that even terrestrial animals from within a food web related to lacustrine environments (e.g., by grazing aquatic plants) can also suffer from a reservoir effect [36].

**Figure 4:**
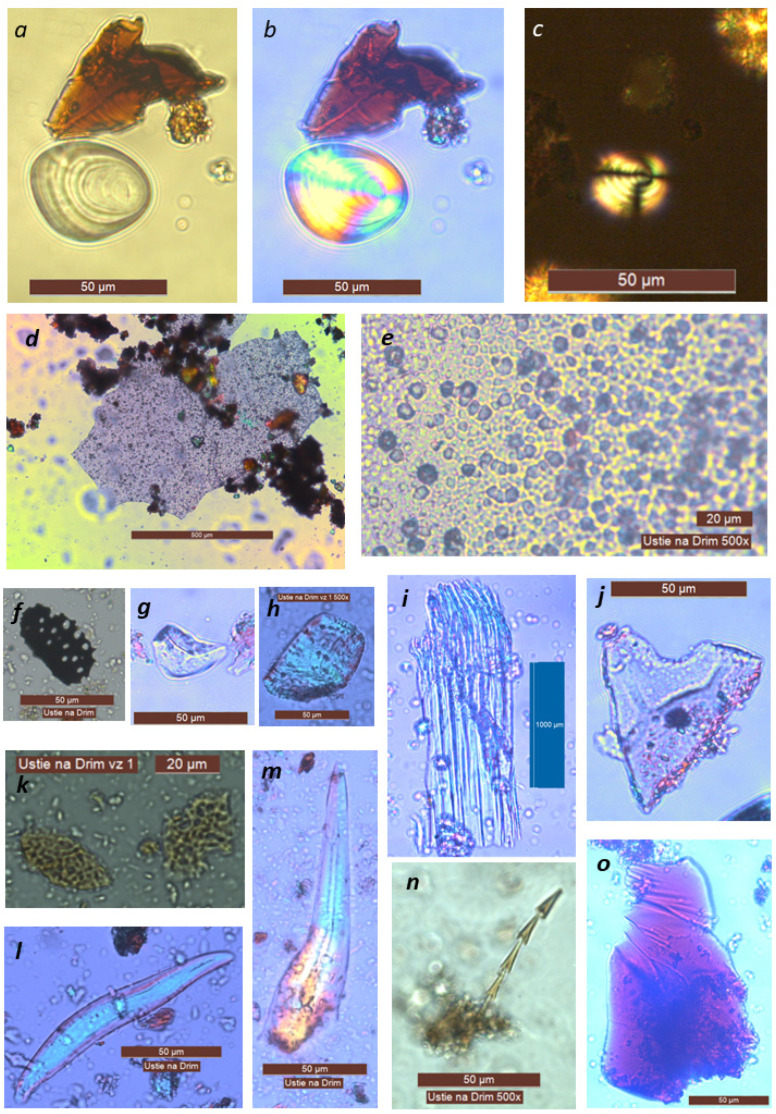
Microresidues in samples SP1 (**h**,**i**,**k**), SP6 (**f**,**l**–**o**) and SP8 (**a**–**e**,**g**,**j**). (**a**–**c**) starch grain, probably the underground storage organ of higher plants, cf. Liliaceae; (**d**,**e**) skeleton consisting of small spheroid psilate phytoliths; (**f**) wood charcoal fragment; (**g**–**k**) phytoliths; (**l**–**o**) other organic residues (probably faunal); (**n**) hair fragment of dermestid beetle larvae, cf. *Trogoderma* sp.) Magnification 500× (except (**d**,**i**)).

**Figure 5:**
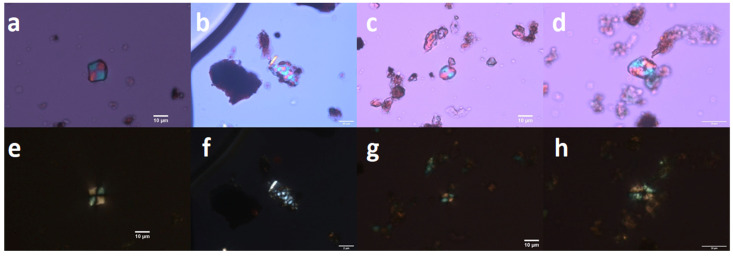
Starch grains of cf. *Setaria* from the ceramic pans KE2, sample SP5 (**a**,**e**); starch grains of cf. *Typha* found in KE4, SP9 (**b**,**f**); starch grains of cf. Poaceae from KE2, SP5 (**c**,**g**); starch grains of cf. *Quercus* in KE2, SP5 (**d**,**h**). Images were taken in visible light (**a**–**d**) and in cross-polarized light (**e**–**h**).

Several types of microscopic organic particles were obtained along with several amorphous clusters of various different materials. The first type of obtained micro-residuals were phytoliths, starch grains, and faunal remains (Figure 4 and Figure 5, Table 1). The most abundant (but still scarce, because generally, the amount of material gained for the microscopy was very low) were phytoliths named spheroid psilate aggregate (Figure 4d,e), which were observed in almost every sample. Some skeletons (aggregates) or single cells of a polyhedral shape were recorded (Figure 4g,j), as well as a particle reminding one of an elongate psilate skeleton (Figure 4i), but appearing blue in the cross-polarized light; hence its plant origin was not particularly certain. A few phytoliths could perhaps be attributed to silicified vessel elements. Polyhedral epidermal cells are the most common type of dicot phytoliths; they are formed in the leaves of many deciduous trees as well as produced by many of the studied herbaceous dicotyledons [39]. The spheroid psilate morphotype often arise as vesicular infillings of the epidermal and parenchyma cells of foliage and reproductive organs in a wide range of dicots, monocots, and some gymnosperms [40]. Note that none of the observed phytoliths could be attributed to cereal remains. The phytoliths which would undoubtedly point to the Poaceae family were not found at all.

Starch as a natural substance is subject to destruction. Starch grains can be damaged mechanically (by breaking), chemically (by the action of acids), enzymatically (by amylase), or by heat (food preparation and cooking). Due to the effect of higher temperatures (above 50–70 °C) on starch grains, especially in humid environments, the gelatinization process can begin. Starch granules gain in volume and lose their typical properties. If the damage is extensive, the morphological identification method cannot be used [41]. Starch is also affected by enzymatic activity, especially amylase from the glycosidases group of enzymes. The source of amylase can be animals, plants, and microorganisms [42]. Despite a number of factors, which may damage or destroy the starch grains, it is possible to find starch grains undamaged or in a state which allows microscopic analysis. As the works of Henry et al. (2009) and Crowther (2012) showed, concerning cooked starchy foods, it is common to observe a variety of reactions to cooking where some granules will appear completely unaffected, while other starch will be partially or fully gelatinized [43,44,45]. An analysis of starch remains was conducted on nine samples. In sample KE1-SP1 one small, round starch granule and an undefined starch were found. In sample KE1-SP2 an oval starch grain, undefined (cf. Poaceae), was found. In samples KE2-SP3 and KE2-SP4, the finding was negative. In contrast, sample KE2-SP5 was rich in starch grains (Figure 5). In this sample following objects were found: an oval starch grain, undefined (cf. Poaceae); a small, round, undefined starch (cf. Poaceae); an oval starch grain, damaged and undefined; a round, damaged, and undefined starch; an elongated atypical starch (cf. *Quercus*) [46,47], and a square-shaped starch (cf. *Setaria*, [46,48]). In the KE3-SP6 sample, a round, undefined starch (cf. Poaceae) was found. Sample KE4-SP8 contained a trapezoid, starch shape with growth rings (cf. Liliaceae) [49]. The last sample, KE4-SP9, contained a round undefined starch (cf. Poaceae) and a cluster of starch grains (cf. *Typha*) [50]. A microscopic investigation was further conducted to reveal the remains of animal structures. The most interesting finding was a fragment of hair belonging to a larval segment of Coleoptera of the family Dermestidae, cf. *Trogoderma* sp. [51] (Figure 4n). This ‘hair’ was identified thanks to its peculiar microstructure; hastisetae (or hastate setae), located on the dorsolateral surface of the tergites of larvae and pupae, are generally quite small (estimated length between 150 and 900 µm) and inserted in setal sockets on the integument trough a pedicel. Hastisetae microstructure consists of two main parts: the shaft and the apical head (Figure 4n). The shaft is made by repeated modules constituted by one cylindrical segment provided with one wreath of spines/scales posterolaterally oriented in the distal part. The head of the seta is a subconical anchor-like structure subdivided into five to seven longitudinal elements; the apex of the head is blunt. This arrow-shaped hair residue is already known from archaeological samples as sporadic non-pollen palynomorphs from the Early Bronze Age to Medieval contexts in Georgia [52,53] and generally occurs in stored animal or plant products.

The identification of starch cf. Poaceae and cf. *Setaria* does not mean the detection of Cerealia on a microscopic level; however, in an archaeological context, their presence on cooking artifacts of this Late Neolithic site allows the possibility of interpreting these findings as to the presence and use of flour from both domesticated and wild plants. Interestingly, the larval hair of a dermestid beetle (cf. *Trogoderma* sp., a genus to which pests of stored plant and animal products belong) was found coincidentally with the above-mentioned types of starch. This combination led to the possible identification of the *Trogoderma granarium* species (khapra beetle) in the record. This insect is generally known as a grain pest; in its larval stage, it is a voracious feeder of stored grains and regularly occurs mainly in cereals, pulses, and their products [54]. The starch grains found from cf. *Typha* and cf. *Quercus* revealed the use of wild plant foods. Both plants are known to be used as food resources for Palaeolithic and Mesolithic hunter-gatherers [55,56,57,58]. Cattail (*Typha* sp.) represents an extraordinarily versatile plant with many uses, such as for roof thatching and making mats or baskets. Moreover, many of its parts, such as the rhizomes, young stems, flower spikes, or pollen of this plant, are edible and are widely known to be used for human consumption [59]. The occurrence of cattail starch in a pan can most likely be explained by its facility for being ground into a nutritive and tasty flour-like cereal flour [60]. Furthermore, acorns have always been an attractive food resource within various resource strategies, including that of agrarian societies. Acorns in prehistoric agricultural communities may have played a role as a food substitute or as a reserve for times of crop failure [61,62,63]. 

The combination of advanced chemical analysis with microscopic evaluation of the microremains brings new evidence about the life of prehistoric people. We propose that the studied ceramic pans were used for the preparation of meals containing meat from common livestock in combination with cereals and wild plants.

## 3. Materials and Methods

### 3.1. Archaeological Samples

**Figure 6:**
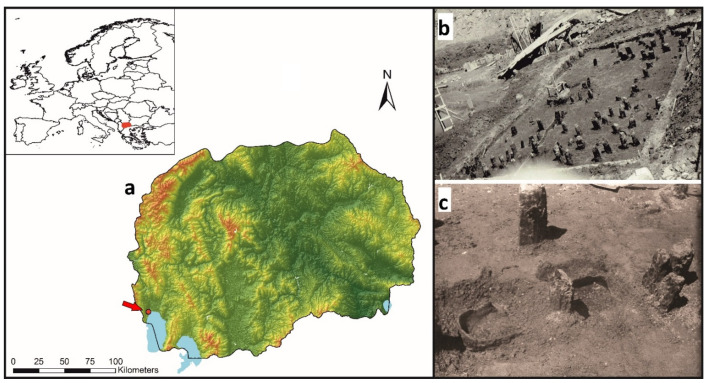
Location of Ohrid area and Ustie na Drim in the North Macedonia (**a**), with archive pictures from the excavation of the palafitte site in 1961 (**b**,**c**). The large archaeological trench with wooden poles (**b**), and the site with an in situ ceramic pan (**c**).

The Ustie na Drim site is located to the immediate north of Lake Ohrid along the banks of the river Crn Drim in Struga (Figure 6). The site is situated in a north-south direction along the riverbed. It is registered in the archaeological map of the Republic of Macedonia as a palafitte settlement from prehistoric times [64]. The initiative for the research of this locality started during some work on regulating the riverbed in 1961, when stone, flint, and bone tools, as well as ceramic fragments, came to the surface. This was reason enough to start protective archaeological excavations. The excavation was conducted over a short period in 1962. Three trenches were opened (trench I with dimensions of 6 × 12 m, trench II with dimensions of 2 × 4 m, and trench III with dimensions of 2 × 2 m) with a total open area of 84 m^2^ [65,66]. Researchers solved the problem of the inflowing river water by constructing a canal along the left bank of the river and smaller canals along the excavated area in the southern and western profile where the water accumulated. Such a remedy allowed digging to continue in relatively dry conditions. The strategy was to excavate in mechanical layers of 15 to 20 cm. In the third mechanical layer, the team came across a deposit of charred and non-charred wooden planks. The situation was interpreted as constructions that had never been destroyed by floods. In the next two mechanical layers, archaeological finds of stone artifacts, bone tools, and pottery were made, together with wooden piles belonging to a Late Neolithic settlement (Figure 6b,c). 

Among the ceramic material, the most common forms were the large, flat, elongated pans (тави), which are shallow oval vessels with pits in the flat bottom. Most of them belonged to a group of ‘rough pottery’ with a dark grey surface and were probably made for some special use [67]. Similar ceramic forms have been found on the Stranata site, named “crepni/црепни”; these are round shallow vessels with a flat bottom, but here without any fingerprints. These vessels have large dimensions and usually a rough texture [68,69]. Outside of today’s borders of the Republic of North Macedonia, a similar ceramic form has been found on the site of Barç, in the Korçë region of Albania [70]. The mentioned sites correspond, according to the relative chronology based on pottery typology, with the second phase of the Late Neolithic in Pelagonia and with the relative chronological position of the Vinca-Tordos II phase or during the Vinca B2 phase in the Central Balkans and the Arapi and Otzaki phases of the Dimini [70,71,72]. This chronological horizon could be dated back to around 5200–5000 BC. 

### 3.2. Residual Sample Extraction

The ceramic fragments from the archaeological research in 1962 were stored at the Struga Museum. As a coarse ceramic, there was still sediment on their surfaces. In 2019, ceramic fragments were sampled for their bioarchaeological residuals and chemistry by a research team from the University of South Bohemia. The list of analyzed samples from ceramic pans are listed in Table 1. It must be pointed out that the pits or depressions made by fingers into the vessel’s surface are technical elements, which were fabricated before the ceramic was fired in the kiln. This technological step with subsequent firing completely eliminates all organic compounds in the ceramic material along with any contamination. On the other hand, the pits were ‘traps’ for residual organic material in the process of baking food. A special case was the fragment of the organic mass from the already-reconstructed pan 7 (Figure 1a). This organic mass fragment was provided in a zip-lock bag separately.

Sampling was performed in two ways (Figure 1). First, the material was scraped from the finger pits with a scalpel directly to polypropylene 2 mL microcentrifuge tubes (series KE1-7, positions KE1-1, KE1-2, and KEx-y). The second series of sampling was processed by liquid extraction using distilled water (sub-series SP for KE1-4, see below). Samples were stored in a refrigerator and transported to the laboratories.

### 3.3. Sampling for Starch, Phytoliths, and Non-Pollen Objects

A micro-pipetting method was used to sample starch grains from the ceramic fragments. A small quantity of distilled water (approximately 100 μL) was placed directly onto the surface of the examined object or pores in the structure of the ceramic pans. Consequently, the water drop containing the extracted residues was collected with a pipette set to 20 μL. Samples were stored in microtubes with an ethanol solution. A drop of the sample was placed on a slide and covered with a coverslip. The corners of the coverslip were fixed with clear nail polish, and samples were left to dry. A drop of distilled water was added to the dried sample before microscopy. Identification was accomplished by direct observation (using a microscope Leica DM2500 P) and comparison with specimens from a reference collection [73,74,75,76]. The analysis of starch grains was based on the microscopic observation of samples in polarized and non-polarized light. The structures found in the samples taken from the archaeological artifacts (ceramic pans) were recognized on the basis of their optical properties and the morphological features of the starch grains.

Furthermore, an analysis of phytoliths and non-pollen objects was performed. Samples in microtubes, originally mounted in ethanol, were transferred to distilled water, left to sedimentation overnight, and after pipetting off the water, the pellet was dried in a laboratory drier at 50 °C (4 h). Then 1 mL of a heavy liquid (sodium polytungstate, SPT) calibrated at a density of 2.35 g·m^−3^ was added to every tube in order to separate phytoliths and other less dense organics from mineral particles. Samples were centrifuged at 800 rpm for 5 min. After that, the supernatant with its floating fraction was transported to new tubes. Subsequently, 2 mL of distilled water was added, and the samples were centrifuged three times (3 min/1500 rpm) to clean the SPT residue. Residues were mounted in distilled water and observed by a Leica DM2500P polarizing microscope (with attached camera). Whenever possible, phytoliths were named following the International Code for Phytolith Nomenclature ICPN 2.0 [38]. Organic matter from the reconstructed KE7 basin was observed using a Keyence VHX 7000 digital microscope (Figure 3). Part of sample KE7 (and KE4-2) was analyzed by radiocarbon dating at the University of Georgia, Center for Applied Isotope Studies using the CAIS 0.5 MeV accelerator mass spectrometer.

### 3.4. Gas Chromatography/Mass Spectrometry (GC/MS)

Gas Chromatography/Mass Spectrometry (GC/MS) was used for the determination of the semi-polar and nonpolar compounds in the acetone/chloroform extract sample [21]. Briefly, the solid material was taken from the inner part of ceramic pans, and 16–50 mg of the material was directly extracted using 1 mL acetone/chloroform solution (50:50, *v*/*v*). Note that from each ceramic pan, the weight of the sample for analysis was the same. After centrifugation (4400 RPM), the liquid part was transferred to a 1.5 mL glass vial, dried by a fine stream of nitrogen, and consequently derivatized using 20 μL *N*,*O*-bis(trimethylsilyl)trifluoroacetamide (Sigma-Aldrich, St. Louis, MO, USA) and 20 μL pyridine (HPLC grade, Sigma-Aldrich, St. Louis, MO, USA). The measurement was performed by an Agilent 7010 Triple Quadrupole GC/MS system with Mass Hunter software (Agilent Technologies, Palo Alto, CA, USA). The separation was performed on two (5% Phenyl)-methylpolysiloxane HP 5 ms Ultra Inert capillary columns connected in a series (15 m × 0.25 mm × 0.25 µm, each) with a constant flow of 1.0 and 1.2 mL min^−1^, respectively. Nitrogen (N2 4.8. Messer Group GmbH, Germany) was used as a collision gas with a flow rate of 1.5 mL min^−1^ and helium (He 5.0. Siad, Italy) as a quench gas with a flow rate of 2.25 mL min^−1^. The initial oven temperature was 70 °C for 5 min; then, the oven was heated at a rate of 15 °C min^−1^ to the value of 320 °C, which was held for 10 min. The injection volume of the extracts was 1 µL with splitless injection. The identification of the compounds was made using the NIST 14 library. Comparison and quantification were made using the authentic standard of cholesterol (Sigma-Aldrich, St. Louis, MO, USA).

### 3.5. Enzyme-Linked Immunosorbent Assay (ELISA)

For the determination of the animal species of organic residues (proteins), an ELISA was used [28,77]. BioKits for Speciation and Identification allowed for the distinguishing between beef, pork, poultry, and mutton proteins. This ELISA used microwell modules and thermostable species-specific muscle proteins. It is a non-competitive, sandwich-type assay (Neogen, Lansing, MI, USA) and was used according to the manufacturer’s instructions with some changes, such as a lower volume of the sample as available in the archaeological material [28]. Additionally, aside from the instructions, samples were not boiled due to the fact that proteins in the archaeological objects could have been heat-treated in the past. In the case of the test for the presence of pork, the samples were boiled for the second replication and compared with the results from the previous (the first) replication; thus, possible contamination from the non-boiled proteins could be detected. Contamination by non-boiled proteins is usually caused by midgut mucosa that is present in animal feces [29].

## 4. Conclusions

The advanced chemical and microremains analyses of ceramic pans from the Ustie na Drim site have provided multiple evidence of their artifact use in the past. The location of the sampled material in pits on the pan surface and the character of the mass directly joined with the artifact surface reduces the possibility of contamination. Overall, the record is fragmentary; however, each of the methods used contributed to answering the basic question of what was usually prepared for a meal in this kind of pottery. Concerning the stratigraphic position of these artifacts, it was possible to determine their age by relative chronology to around 5200 BC. Two samples from the surface of the pans were dated by AMS ^14^C. Both radiocarbon dates were probably affected by an intake of old carbon from the environment. Considering the estimated reservoir effect of Lake Ohrid, both dates indicate an origin for the organic mass within the period of functional use of the ceramic pans. Since radiocarbon dates are older than the relative chronology of pans, we can exclude any post-depositional contamination by material from a later archaeological period. The most relevant result was given by the advanced chemical analysis. The main compound that occurred in all samples is cholesterol. A major source of cholesterol is animal fat and meat (its presence was proved by immunological test). Recent data indicate the amount of cholesterol in meat was roughly 75 mg/100 g (fatty parts like liver, brains, etc., contain a much greater content, up to several hundred milligrams per 100 g) [78]. It should be noted that the concentration of cholesterol in the samples of organic residues from the ceramic pans’ pits was significantly higher than in the reference samples, a fact that excludes the possibility of cross-contamination of a pan’s pit by the surrounding material at the storage location. The presence of denatured proteins in the ceramic pan was also confirmed using an ELISA. Based on the results, we suppose that the analyzed ceramic pans from Ustie na Drim were used for the preparation of meals containing meat from common livestock in combination with cereals and wild plants.

## Figures and Tables

**Table 1:** List of studied samples with the focus on the concentration of cholesterol, starch grains, phytolith, and non-pollen objects. SP: sample was taken for microscopical evaluation of microremains; positive: microobject was found in the prepared sample; negative: no microobject was found).

Artefact	Sample	Concentration of Cholesterol (mg/g)	Phytoliths and Non-Pollen Objects	Starch	Position of Sample in Ceramic Pan
KE1	1	0.05	-	-	pit (residue)
KE1	2	0.08	-	-	pit (ceramic under KE1-1)
KE1	3	0.00	-	-	edge, the reference sample
KE1	SP1	-	positive	positive	pit
KE1	SP2	-	-	positive	edge
KE2	1	0.03	-	-	pit (residue)
KE2	2	0.01	-	-	pit (ceramic under KE2-1)
KE2	3	0.92	-	-	pit (residue)
KE2	4	0.04	-	-	pit (ceramic under KE2-3)
KE2	5	0.00	-	-	bottom, the reference sample
KE2	6	0.00	-	-	inner surface (organic temper)
KE2	7	0.01	-	-	surface (the reference sample)
KE2	SP3	-	-	negative	pit
KE2	SP4	-	-	-	pit
KE2	SP5	-	-	positive	edge
KE3	1	0.02	-	-	inner surface (close bottom part, baked mass)
KE3	2	0.13	-	-	inner surface (under edge, baked mass)
KE3	3	0.01	-	-	inner surface (ceramic under KE3-1)
KE3	4	0.34	-	-	inner surface (ceramic under KE3-2)
KE3	5	0.04	-	-	edge (the reference sample)
KE3	SP6	-	positive	positive	pit
KE3	SP7	-		negative	pit
KE4	1	0.44	-	-	inner edge (close bottom part, baked layer)
KE4	2	0.66	-	-	inner edge (close upper part, baked layer)
KE4	3	0.19	-	-	inner edge (ceramic under KE4-1)
KE4	4	0.46	-	-	inner edge (ceramic under KE4-2)
KE4	5	0.01	-	-	edge (the reference sample)
KE4	SP8	-	positive	positive	pit
KE4	SP9	-	-	positive	pit
KE5	1	0.09	-	-	inner edge (close bottom part, thin layer)
KE5	2	0.24	-	-	inner edge (close to KE5-1)
KE5	3	0.02	-	-	inner edge (ceramic under KE5-1)
KE5	4	0.16	-	-	inner edge (ceramic under KE5-2)
KE5	5	0.01	-	-	edge (the reference sample)
KE6	1	0.00	-	-	wall (close upper part, baked mass)
KE6	2	0.02	-	-	wall (close to KE6-1
KE6	3	0.00	-	-	edge (the reference sample)
KE7	1	0.17	-	-	organic residue (taken before conservation)

**Table 2:** Radiocarbon dates from organic residuals. Calibrated in OxCal 4.4 software using IntCal20 calibration curve [37,38].

Sample	Lab. Code	BP Age	cal BC 68.4%	cal BC 95.4%
KE 4-2	UGAMS-49232	7370 ± 30	6341–6313 (13.2%)	6369–6297 (22.6%)
			6260–6216 (31.3%)	6269–8209 (35.6%)
			6142–6092 (23.7%)	6198–6085 (37.2%)
KE 7	UGAMS-49233	7480 ± 25	6411–6366 (36.3%)	6422–6332 (53.3%)
			6308–6265 (32.0%)	6319–6319 (42.1%)

## Data Availability

The data presented in this study are available on request from the corresponding author. The data are not publicly available due to the privacy policy of the author’s institution.

## Supplementary Materials

The following are available online, Figure S1: Total ion current (TIC) chromatogram of sample KE4-2 (green line; inner edge: close upper part, baked layer) and KE4-5 (orange line; edge: the reference sample). Table S1: Table of the most significant compound detected in sample KE4-2.

## Sample Availability

Studied samples are not available.
