# Supplementary material for: What about Dinner? Chemical and Microresidue Analysis Reveals the Function of Late Neolithic Ceramic Pans
Source: Molecules. 2021 Jun 3;26(11):3391. doi: 10.3390/molecules26113391 (PMC8199953; doi:10.3390/molecules26113391)
Supplement: Supplementary file 1 [file molecules-26-03391-s001.zip › molecules-1241678-supplementary.pdf]

What about dinner? Chemical and microresidual analysis reveal the function of the Late Neolithic ceramic pans

Jaromír Beneš, Valentina Todoroska, Kristýna Budilová, Jaromír Kovárník, Jaroslav Pavelka, Nevenka Atanasoska, Jiří Bumerl, Assunta Florenzano, Tereza Majerovičová, Václav Vondrovský[6], Michaela Ptáková, Petr Bednář, Lukáš Richtera and Lukáš Kučera

Figure S1: Total ion current (TIC) chromatogram of sample KE4-2 (green line; inner edge - close upper part, baked layer) and KE4-5 (orange line; edge - the reference sample). List of identified compounds is shown in Supplement 1B.

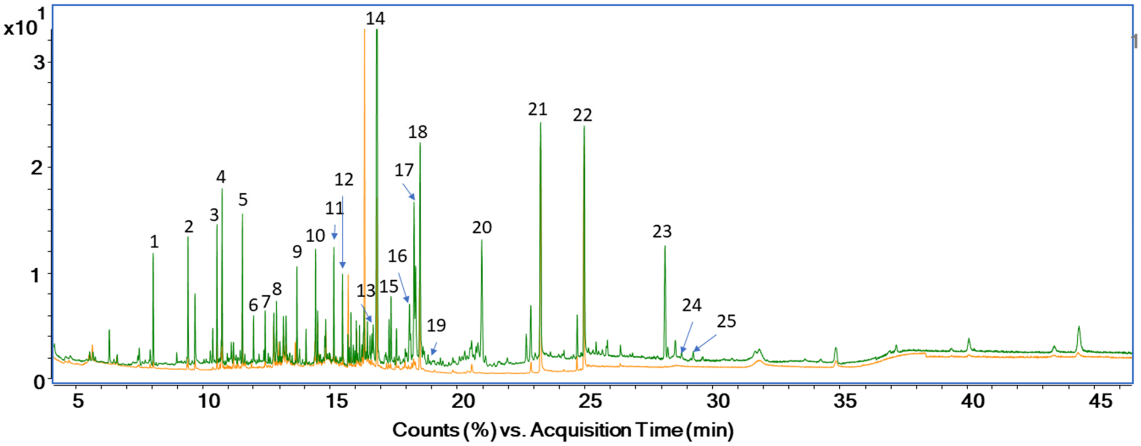

Table S1: Table of the most significant compound detected in sample KE4-2

Peak #	RT (min)	Compound
1	8.05	Hexanoic acid, TMS derivative
2	9.42	Heptanoic acid, TMS derivative
3	10.55	Octanoic acid, TMS derivative
4	10.75	Glycerol, 3TMS derivative
5	11.54	7-Metyloctanoic acid, TMS derivative
6	11.98	Pentanedioic acid, TMS derivative
7	12.44	Decanoic acid, TMS derivative
8	12.89	Hexanedioic acid, TMS derivative
9	13.69	3-Methyladipic acid, 2TMS derivative
10	14.43	Suberic acid, 2TMS derivative
11	15.15	Azelaic acid, 2TMS derivative
12	15.49	Myristic acid, TMS derivative
13	16.65	18-norabietane
14	16.84	Palmitic acid, TMS derivative
15	17.38	Heptadecanoic acid, TMS derivative
16	18.11	3,7-Dimethyloctanoic acid, TMS derivative
17	18.28	Oleic acid, TMS derivative
18	18.53	Stearic acid, TMS derivative
19	18.64	Retene
20	20.96	Sebatic acid, 2TMS derivative
21	23.26	1-Monopaltmitin, 2TMS derivative
22	24.97	Glycerol monostearate, 2TMS derivative
23	28.15	Cholesterol, TMS derivative
24	28.80	Cholesta-3,5-dien-7-one
25	29.26	Cholest-4-en-3-one
